# Pathophysiology of the inner ear after blast injury caused by laser-induced shock wave

**DOI:** 10.1038/srep31754

**Published:** 2016-08-17

**Authors:** Katsuki Niwa, Kunio Mizutari, Toshiyasu Matsui, Takaomi Kurioka, Takeshi Matsunobu, Satoko Kawauchi, Yasushi Satoh, Shunichi Sato, Akihiro Shiotani, Yasushi Kobayashi

**Affiliations:** 1 Department of Anatomy and Neurobiology, National Defense Medical College, Tokorozawa, 359-8513, Japan; 2 Department of Otolaryngology, Head and Neck Surgery, National Defense Medical College, Tokorozawa, 359-8513, Japan; 3 Department of Biomedical Information Science, National Defense Medical College, Tokorozawa, 359-8513, Japan; 4 Department of Pharmacology, National Defense Medical College, Tokorozawa, 359-8513, Japan

## Abstract

The ear is the organ that is most sensitive to blast overpressure, and ear damage is most frequently seen after blast exposure. Blast overpressure to the ear results in sensorineural hearing loss, which is untreatable and is often associated with a decline in the quality of life. In this study, we used a rat model to demonstrate the pathophysiological and structural changes in the inner ear that replicate pure sensorineural hearing loss associated with blast injury using laser-induced shock wave (LISW) without any conductive hearing loss. Our results indicate that threshold elevation of the auditory brainstem response (ABR) after blast exposure was primarily caused by outer hair cell dysfunction induced by stereociliary bundle disruption. The bundle disruption pattern was unique; disturbed stereocilia were mostly observed in the outermost row, whereas those in the inner and middle rows stereocilia remained intact. In addition, the ABR examination showed a reduction in wave I amplitude without elevation of the threshold in the lower energy exposure group. This phenomenon was caused by loss of the synaptic ribbon. This type of hearing dysfunction has recently been described as hidden hearing loss caused by cochlear neuropathy, which is associated with tinnitus or hyperacusis.

In recent years, blast injury is a critical issue in hearing research because of the increase in terrorist acts that employ improvised explosive devices both in civilian situations and wars such as those in Afghanistan or Iraq^1^,^2^. After an explosive detonation, high-pressure gasses expand away from the center of the explosion, compressing the surrounding air and producing blasts. The blast pressure wave exerts forces mainly at air-tissue interfaces of the body; thus, the auditory system is a high-risk organ^3^. The auditory system (i.e., the tympanic membrane, cochlea, and central auditory pathways) is the organ that is most commonly damaged by blast overpressure^1^,^4^,^5^. For example, tympanic membrane perforation (TMP), temporary and permanent hearing loss, tinnitus, and hyperacusis were reported as consequences from blast exposure after the April 15, 2013 Boston Marathon bombings^6^. As the tympanic membrane is the most sensitive to overpressure, TMP is the most frequent symptom seen after severe blast exposure^7^,^8^. Although the sensorineural component of hearing disability is relatively mild compared with conductive hearing loss such as TMP, its incidence rate is high in blast-injured patients^9^,^10^. The permanent sensorineural hearing loss (SNHL) caused by blast exposure is untreatable and is often associated with a decline in the quality of life^11^,^12^. In contrast, conductive hearing loss is usually treatable by surgery and other therapeutic means. Although several models for blast-induced hearing loss have been developed recently, reports of animal models of blast-induced SNHL are scarce, and the mechanisms have not been adequately investigated. Recent reports demonstrated permanent hearing loss in a mouse model using a blast chamber^13^,^14^; however, the cochlear pathology of this model was severer than the human hearing disability reported in real situations such as after the Boston Marathon bombings^6^. In addition, this blast-induced mouse hearing loss model most likely also involves TMP; thus, detailed sensorineural hearing function cannot be tested.

In this study, we analyzed physiological and structural changes in the inner ear of a rat model that replicates pure SNHL induced by blast injury caused by laser-induced shock wave (LISW)^15^,^16^. We show that SNHL after blast exposure was primarily caused by stereociliary bundle disruption of the outer hair cells (OHCs). In addition, a decrease in the number of synaptic ribbons in the inner hair cells (IHCs) and spiral ganglion cells (SGCs) was associated with so-called “hidden hearing loss,” which is implicated in tinnitus and hyperacusis (diminished sound-level tolerance).

## Results

### Shock wave energy-dependence of sensorineuronal hearing dysfunction

**Figure 1:**
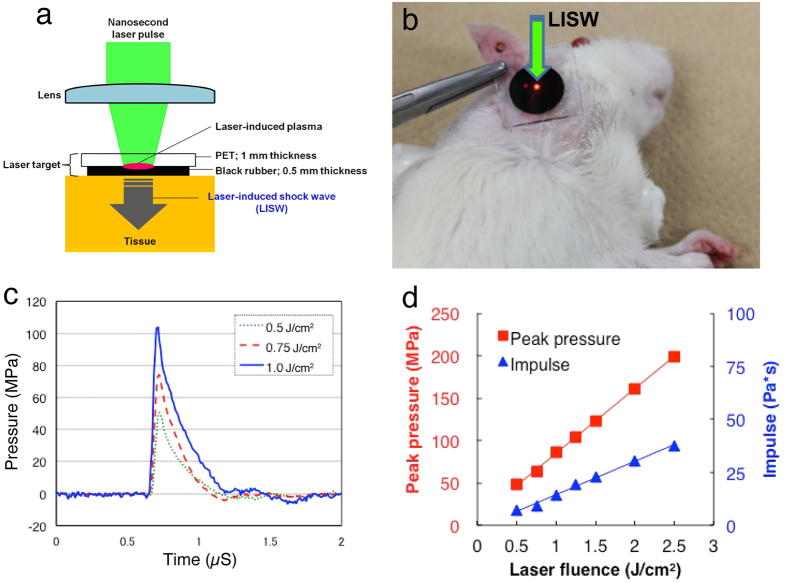
Generation and characteristics of LISW. (**a**) Experimental setup for the LISW. Shock waves were generated by irradiating a laser target (black rubber) with a Q-switched Nd:YAG laser. Plasma formation occurred at the interface between a transparent material and the black rubber. (**b**) Image showing how to apply an LISW to induce inner ear injuries. (**c**) Typical temporal waveforms of LISWs generated at different laser fluences on the laser target. (**d**) Dependence of peak pressure and LISW impulse on laser fluence. LISW, laser-induced shock wave.

We first conducted several complementary tests for assessing inner ear function, ABR threshold, ABR wave I amplitude, and DPOAE measurements. ABR is sound-evoked potentials in the ascending auditory pathways, and the first ABR wave (wave I) represents the summed activity of the cochlear nerve^17^. DPOAE can directly measure the OHC function, which is a biological motor for amplifying the motion of the sensory epithelium^18^. LISWs were generated as described previously^15^ (Fig. 1).

**Figure 2:**
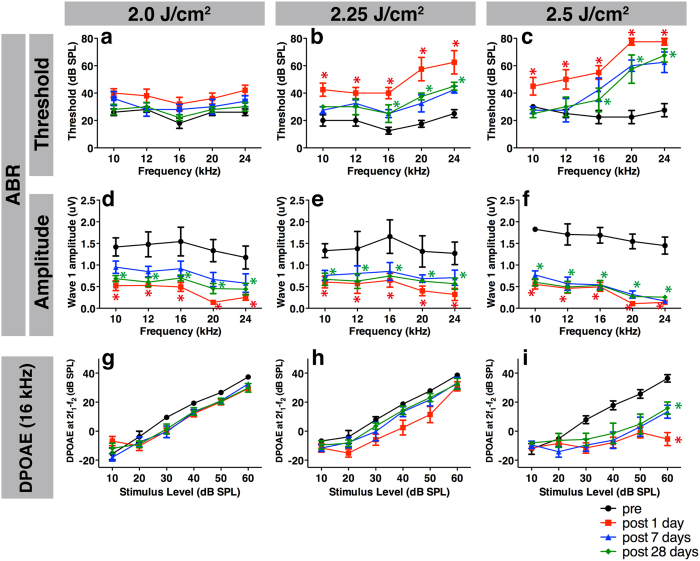
Measurement of hearing function using ABR and DPOAE before and after LISW exposure. (**a**–**c**) Significant ABR threshold shifts one day after LISW (filled squares) exposure were observed up to 1 month after exposure (filled diamonds) in the 2.25 (**b**) and 2.5 J/cm^2^ (**c**) groups compared to the pre-exposure thresholds (filled circles). (**d**–**f**) Despite the absence of ABR threshold elevation, the wave I amplitude was decreased in the 2.0 J/cm2 (**d**) group, which was also seen in the 2.25 (**e**) and 2.5 J/cm^2^ (**f**) groups. (**g**–**i**) The DPOAE level in the 2.5 J/cm^2^ (**i**) group was significantly decreased, whereas no decrease was observed in the 2.0 (**g**) or 2.25 J/cm^2^ (**h**) group. Asterisks indicate significant differences (p < 0.05) compared to the pre-exposure values. Error bars indicate SEs of the means (n = 5 in each group). ABR, auditory brainstem response; DPOAE, distortion product otoacoustic emission; LISW, laser-induced shock wave.

After LISW exposure, the ABR threshold at the exposed ear (right side) was immediately elevated in the high and middle energy groups, with significant elevation remaining up to 28 days after exposure (2.25 and 2.5 J/cm^2^; Fig. 2b,c). In contrast, no elevation was seen in the lower energy group (2.0 J/cm^2^, Fig. 2a). The degree of threshold elevation was energy-dependent. The unexposed ear (left side) showed no significant changes before and after LISW exposure (data not shown). Although the ABR threshold was not elevated in the 2.0 J/cm^2^ group, the ABR wave I amplitude was significantly decreased from immediately after LISW exposure up to 28 days after exposure (Fig. 2d), which was similar to the higher energy groups (Fig. 2e,f). These findings indicate that hearing dysfunction, as indicated by a decrease in sound-evoked potentials in the ascending auditory pathway, was induced by all of the LISW energies we tested.

Next, we conducted DPOAE to evaluate OHC function. DPOAE was only measured at f2 frequencies 10, 12, and 16 kHz (and not 20 and 24 kHz) because the HearID system we used in this study is designed for human clinical use, and 16 kHz is the upper limit. Interestingly, the DPOAE responses at 2.0 and 2.25 J/cm^2^ showed no significant changes (Fig. 2g,h), whereas the DPOAE response at 2.5 J/cm^2^ was significantly decreased immediately after LISW exposure and up to 28 days after exposure (Fig. 2i). At the lower frequencies (10 and 12 kHz), no significant DPOAE changes were observed in any of the energy groups tested (data not shown).

TMP was not observed in the rats after LISW exposure in any energy group. Furthermore, mechanical damage was not observed in the cochelae, such as a round window rupture, stapes dislocation, intracochlear hemorrhage, and a basilar or Reissner’s membrane rupture, under light microscopy using frozen cross sections after LISW exposure (data not shown), which is similar to previous results^16^. These results suggest that the hearing dysfunction observed in this study is caused by damage to the sensorineural component of the auditory pathway.

### LISW reduced the number of synaptic ribbons and spiral ganglion neurons but did not induce hair cell loss

**Figure 3:**
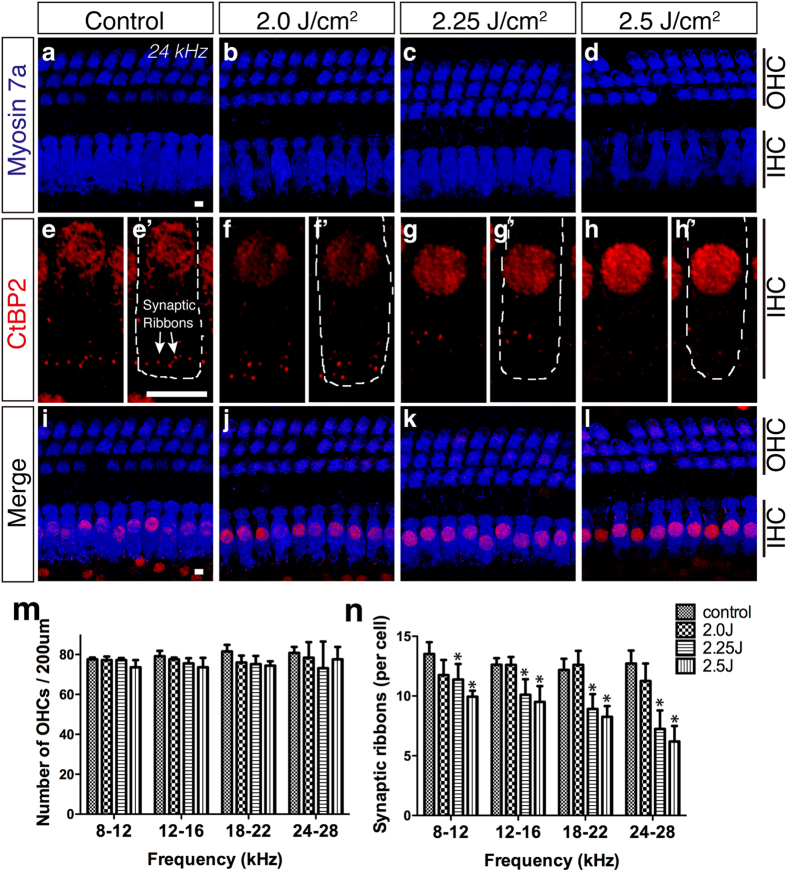
Changes in the organ of Corti after LISW exposure. (**a**–**i**) Confocal images of immunohistochemistry for OHCs (**a**–**d**, blue, anti-myosin 7a), synaptic ribbons (**e**–**h**, red, anti-CtBP2; the white dotted line in e’–h’ shows the contour of the OHCs; anti-CtBP2 also stains the IHC nuclei), and merged images (**i–l**) at 24 kHz. (**m**–**n**) The number of OHCs (**m**) and synaptic ribbons (**n**) 1 month after LISW exposure (n = 5 in each group). Despite ABR threshold shifts observed in the 2.25 and 2.5 J/cm^2^ groups (**c,d**), the number of OHCs remained steady 1 month after LISW exposure (**m**) compared to the control group (**a**). The numbers of synaptic ribbons were decreased by LISW in an energy-dependent manner (**e**–**h**,**n**). Asterisks indicate significant differences (p < 0.05). Error bars indicate SEs of the means. Scale bar is 5 μm. ABR, auditory brainstem response; IHC, inner hair cell; LISW, laser-induced shock wave; OHC, outer hair cell.

To assess the cause of SNHL after LISW exposure, we next conducted surface preparation analysis of the organ of Corti 1 month after LISW exposure. Surprisingly, the number of OHCs (anti-myosin 7a:blue, Fig. 3) in the LISW-exposed ear (right side) did not change at any frequency area in all of the LISW energy groups (Fig. 3a–d,m), although the ABR threshold was elevated in higher LISW energy groups. The number of OHCs in the unexposed ear (left side) and the number IHCs in both ears did not change (data not shown).

Next, we focused on the synapses between IHCs and cochlear nerve fibers. Synaptic ribbons are a characteristic structure of hair cell afferent synapses and are involved in vesicle delivery to the active zone^19^. The number of synaptic ribbons (anti-CtBP2: red, Fig. 3) in the LISW-exposed ear (right side) was significantly decreased in the middle and high energy groups (2.25 and 2.5 J/cm^2^) at all frequencies we tested (Fig. 3e–h,n). The number of synaptic ribbons in the unexposed ear (left side) remained unchanged in all groups (data not shown).

**Figure 4:**
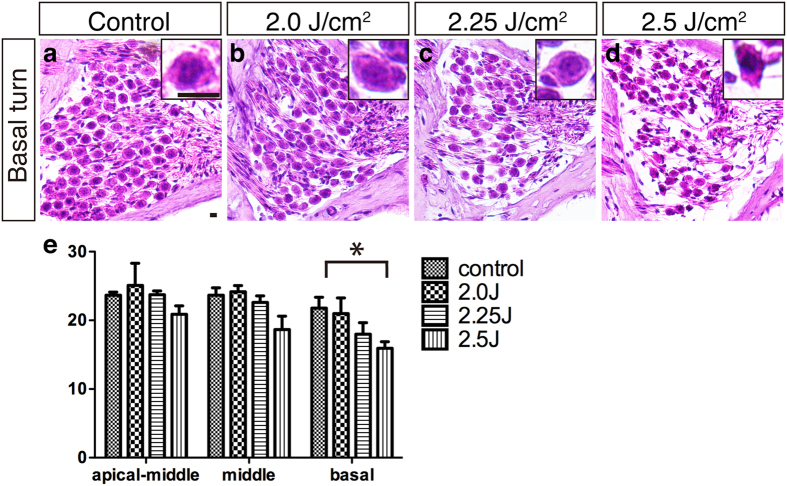
Changes in the spiral ganglion after LISW exposure. (**a**–**d**) Images of the SGCs using HE-stained frozen sections from the cochlear basal turns. Insets show high-power views of a single SGC. (**e**) The number of SGCs 1 month after LISW exposure (n = 5 in each group). The concentration of SGCs at the basal turn in the 2.5 J/cm^2^ group was significantly decreased (**d**,**e**), compared to the control (**a**). The basal turn of the SGC in the 2.25 J/cm^2^ (**d**, inset) group shows shrinking compared to the control (**a**, inset). The asterisk indicates a significant difference (p < 0.05). Error bars indicate SEs of the means. Scale bar is 5 μm. LISW, laser-induced shock wave; SGC, spiral ganglion cell; SE, standard error.

We further analyzed the SGCs at 1 month after LISW exposure using frozen cross sections (Fig. 4). The density of SGCs in the high energy (2.5 J/cm^2^) group in the LISW-exposed ear showed significant reduction, with an atrophic appearance of the remaining cell bodies only at the high frequency area (Fig. 4d,e). The number of SGCs on the unexposed side was unchanged in all groups (data not shown).

These histological findings are compatible with the reduction of ABR wave I amplitude after LISW exposure because synaptic ribbon counts in normal ears provide an accurate metric of the IHC afferent innervation^20^,^21^.

### Stereociliary bundle disruption of the OHCs is the primary cause of the ABR threshold shift in LISW-induced hearing dysfunction

We found an ABR threshold elevation (about 30 dB) up to 1 month after LISW exposure at higher frequencies above 16 kHz in the 2.25 and 2.5 J/cm^2^ groups (Fig. 2b,c). However, this 30-dB threshold shift cannot be explained simply by disordered synapses between the IHCs and SGCs or the reduced number of SGCs we found histologically (Figs 3 and 4). There is extensive evidence concerning the relationship between the number of synaptic ribbons and ABR threshold shifts^21^. Data from this study showed ABR threshold shifts of only a few dB if the number of synaptic ribbons is reduced by 50%. Thus, we conducted scanning electron microscopy to determine the etiology of the ABR threshold elevation.

**Figure 5:**
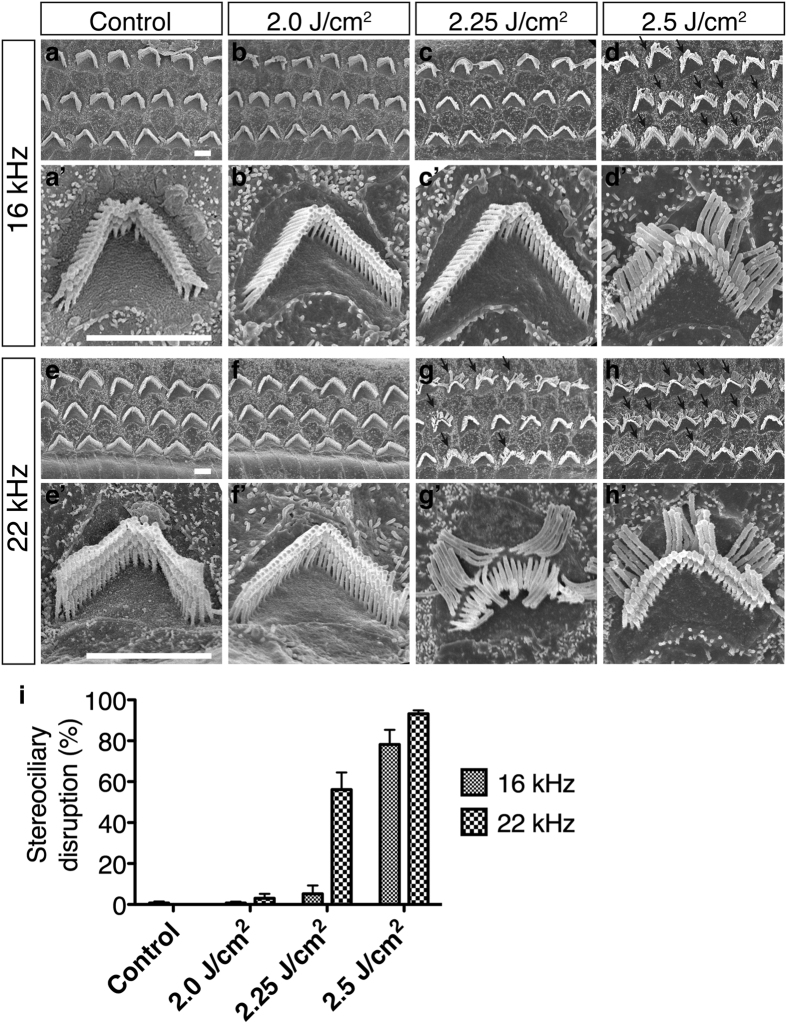
Surface structures in the OHCs. (**a**–**d**) Scanning electron microscopy images of three rows of OHC stereocilia at the 16 kHz organ of Corti area. (a’–d’) high-power views of single OHC stereocilia. (**e**–**h**) and (e’–h’) Images from the 22 kHz organ of Corti area. (**i**) The stereociliary disruption ratio on OHCs one month after LISW exposure (n = 3 in each group). Stereociliary bundle disruption, i.e., the outermost layer of bundles being broken from the root (arrows), was seen in the 2.25 J/cm^2^ (22 kHz area, g and g’) and 2.5 J/cm^2^ groups (16 and 22 kHz area, **d, h,** and d’, h’). Scale bar is 2 μm. Error bars indicate SEs of the means. OHC, outer hair cell; SE, standard error.

Sound-evoked vibration is transduced by stereocilia at the apical surfaces of hair cells^22^,^23^. Normal stereocilia of the OHC have a “V” shape with three bundle rows (Fig. 5a’). However, deformed stereocilia of the outermost row of bundles were bent at their base toward the lateral side (Fig. 5d,g,h arrows). This was seen in the higher energy groups (2.25 J/cm^2^ and 2.5 J/cm^2^), which also exhibited ABR threshold elevation (Fig. 5i). Moreover, the decreased DPOAE found at 16 kHz in the 2.5 J/cm^2^ group (Fig. 2i) may have been directly caused by OHC dysfunction induced by deformation of the stereocilia (Fig. 5h).

## Discussion

### Blast exposure can cause hidden hearing loss

We have demonstrated a novel animal model of SNHL after blast injury using LISW to replicate pure SNHL after blast injury without any conductive hearing loss. On the shock wave setting we used in this study, the degree of hearing loss was relatively mild. The degree of the ABR threshold elevation we observed in this study was very similar to the sensorineural hearing loss in human subjects recovered from blast injuries in real bombing situations^6^. Because conventional blast injury models of hearing loss have unavoidably caused TMP^13^,^14^, it has been impossible to accurately evaluate sensorineural hearing function in these animal models. Moreover, intense blast waves of impulse noise can severely damage the organ of Corti by tearing it loose from the basilar membrane as well as hair-cell loss^24^,^25^, resulting in severe SNHL, which is rarely seen in humans^6^. Therefore, our present model is suitable for analyzing mild SNHL caused by blast injuries. In addition, we observed reduction of the ABR wave I amplitude without elevation of the ABR threshold in the 2.0 J/cm^2^ group; this phenomenon can be attributed to the loss of synaptic ribbons. This type of hearing dysfunction has recently been described as hidden hearing loss^26^,^27^ and is caused by cochlear neuropathy^21^. The pathology of hidden hearing loss is closely related to the pathogenesis of tinnitus^26^,^28^ and hyperacusis^29^. Therefore, our present model is valuable for analyzing the etiologies of tinnitus or hyperacusis, which are the most frequent symptoms after blast injury^6^,^11^,^30^,^31^.

### Stereociliary disturbance without hair cell loss is a unique characteristic of blast-induced hearing dysfunction

Our scanning electron microscopic observation revealed the disturbance of stereocilia in the frequency areas that matched those in the DPOAE reduction even 1 month after LISW exposure. Interestingly, the bundle disruption pattern was unique, with most of the disturbed stereocilia observed in only the outer row, whereas the inner and middle row stereocilia remained intact. Stereociliary disturbance is also observed in noise-induced hearing loss; however, the pattern of stereocilia damage after noise-induced hearing loss is quite different. The pattern has been reported as fused, splayed, or missing stereociliary bundle arrangements because of white noise exposure (110 dB PSL, 2 h)^32^. Surprisingly, this study also reported spontaneous morphological recovery 1 month after noise exposure^32^. Meanwhile, similar stereociliary damage was also reported after noise exposure^33^,^34^. These reports showed that the tallest row of stereocilia was ultrastructurally damaged, especially at the rootlet, after a noise-induced hearing loss model. The phenomenon that the tallest stereocilia row was the most fragile could be explained by the fact that only the tallest OHC stereocilia row insert into the tectorial membrane. In previous blast-induced hearing loss models induced by a blast tube^13^, normal stereociliary bundle morphology was observed in confocal images of the surface of hair cells. Our results were largely similar as those seen in the figure displayed in Cho *et al*.’s study, which showed that the outer row of bundles was bent at their base in confocal images taken after blast exposure. Therefore, we believe that this morphological change in the stereocilia represents a characteristic morphology for OHC exposure to blast overpressure. The exact mechanism of this stereociliary disturbance is unknown. One possible reason for this unique morphological change may be the disconnection of tip links and side links between the disturbed outermost row stereocilia and the middle row stereocilia. Although mammalian stereocilia have no spontaneous regenerative capacity after total disappearance, even if the hair cell itself is still alive^35^, the tip link and side link, which are cadherin23 and protcadherin15, can be remodeled after disconnection in neonatal cochlear expants^36^,^37^. However, in this model, we speculate that the tip link and side link would be mechanically cut by the shock wave and that the distance between the outermost row of stereocilia and the second row would be too far for regeneration of the tip link and side link. Furthermore, rootlets of the outermost row of stereocilia, which consist of actin and bundling proteins such as TRIOBP^38^, would also be mechanically damaged.

### LISW can replicate blast-induced organ damage

When an explosion occurs, the shock wave is followed by a blast wind that is faster than sound. Therefore, a blast wave consists of blast wind and a shock wave. Either of the blast wave components can affect living tissue; however, the shock wave component, which is characterized by an extremely fast rise in pressure and high peak pressure, is considered to be the most invasive^15^. A shock wave can be induced by irradiation of a solid material with a high-energy laser pulse; the resulting shock wave is called a photomechanical wave or LISW. Previous studies have developed animal models of blast-induced traumatic brain injury^15^, pulmonary injury^39^, and inner ear injury^16^ by utilizing LISW. The most significant advantage of LISW for blast-related research is site specificity for the injured organ, because LISW does not affect areas outside the targeted site in exposed animals. Using this characteristic, we were able to avoid TMP in our present model. For blast-induced hearing research, this enables the creation of complete one-sided hearing dysfunction because the contralateral ear is not exposed to the LISW. For example, it is known that complete single-side light to moderate hearing dysfunction is ideal for conducting the tinnitus behavior test in rodents^40^. The controllability of LISW output energy is also a beneficial advantage for blast-related research. Previous reports of LISW-induced hearing loss models showed severe and permanent hearing loss accompanied with severe hair cell loss utilizing a ruby LISW, which is able to generate much greater energy levels than those that we used in this study. We could easily adjust the LISW output level to replicate the severity of SNHL that is frequently seen in clinical situations. Because of these advantages, this LISW-induced hearing dysfunction model would be an ideal platform to analyze the etiology of blast-induced hearing loss and to establish novel strategies for treating blast injuries.

## Methods

### Animals

Thirty-six Sprague Dawley rats (male, 35–42 days old) weighing 150–200 g with normal Preyer’s reflex were purchased from Japan SLC (Hamamatsu, Shizuoka, Japan). The animals were given free access to water and were fed a regular diet. They were individually housed and maintained at 23 °C–25 °C. All experimental procedures reported in this study were approved by the Institutional Animal Care and Use Committee of the National Defense Medical College, and were in accordance with the guidelines of the National Institutes of Health, and the Ministry of Education, Culture, Sports, Science and Technology of Japan. All efforts were made to minimize the number of animals used and their suffering.

### Inner ear exposure to LISW

LISWs were generated as described previously^15^ (Fig. 1). Briefly, LISWs were generated by irradiating a laser target with a 532-nm Q-switched Nd: YAG laser (Brilliant b, Quintal; pulse width, 6 nanoseconds FWHM). The laser target was a 10-mm-diameter, 0.5-mm-thick black natural rubber disk; a 1.0-mm-thick transparent polyethylene terephthalate sheet was bonded to the top of the target to confine the laser-induced plasma and to increase the LISW impulse. The laser pulse was focused with a plano-convex lens to a 4.0-mm diameter spot on the laser target. The outputs of the laser pulses were set at three energy densities (fluences): 2.0 J/cm^2^, 2.25 J/cm^2^, and 2.5 J/cm^2^. Sham exposure was used as the control. The rats were anesthetized by intraperitoneal injection of ketamine (50 mg/kg) and medetomidine (1.0 mg/kg), and their right postauricular regions were carefully shaved to avoid retaining trapped air in their fur. The rats were then fixed on a plate, and the postauricular regions were positioned in the focal area of the LISW. To ensure acoustic impedance matching, ultrasound conductive gel was used between the laser target and the skin surface.

### ABRs and distortion product otoacoustic emissions

The ABRs of all thirty-six rats were measured before and 1 day, 1 week, and 4 weeks after LISW irradiation. Under general anesthesia, stainless steel needle electrodes were placed subcutaneously at the center of vertex and ventrolateral surfaces of the left and right ears for recording. Tone burst stimuli of 1 ms rise/fall time at frequencies of 10, 12, 16, 20, and 24 kHz were generated; the amplitudes were specified by the sound generator and attenuated by a real-time processor and programmable attenuator (RP2.1 and PA5; Tucker-Davis Technologies, Alachua, FL, USA). The sound stimuli were produced by a coupler-type speaker (ES1spc; Bio Research Center, Nagoya, Japan). ABR waveforms were recorded for 12.8 ms at a sampling rate of 40,000 Hz using 50–5,000 Hz band-pass filter settings; waveforms from 512 stimuli at a frequency of 9 Hz were averaged. The ABR waveforms were recorded in descending 5 dB SPL intervals from the maximum amplitude until no waveform could be visualized. The ABR threshold was defined as the lowest stimulus level at which a repeatable wave I could be identified in the response waveform. Wave I of the ABR is defined as the earliest negative–positive deflection in the response waveform and corresponds to the summed activity of cochlear afferents^21^. For calculating wave I amplitude, the peak-to-peak amplitude at 80-dB SPL was computed by offline analysis of stored waveforms.

Distortion product otoacoustic emissions (DPOAEs) at 2f1–f2 were recorded with the HearID system (Mimosa Acoustics, Champaign, IL, USA) in a soundproof room. The f2/f1 ratio of the primary tones was set to 1.2, and the DPOAE input/output functions were measured at f2 frequencies of 10, 12, and 16 kHz. The L1 always presented +10 dB above the L2. The animals were anesthetized with the ketamine and medetomidine mixture, as described above. The probe was placed in the animal’s external ear canal. Input/output functions were obtained by increasing the L1 intensity from 20 to 70 dB SPL at f2 frequencies of 10, 12, and 16 kHz. DPOAEs were recorded before and 1 day, 1 week, and 4 weeks after LISW irradiation. The DPOAE threshold was defined as the f1 level required to produce a DPOAE of 0-dB SPL.

### Immunohistochemistry

All histological examinations were conducted following the final ABR assessment (1 month after LISW exposure). For whole mount preparation, the rats were perfused transcardially with heparinized PBS followed by 500 ml 4% paraformaldehyde (PFA) in 0.1 M phosphate buffer (PB) at room temperature (20 °C–25 °C) under general anesthesia. After decapitation, the cochleae were quickly dissected out and small openings were made at the round window, oval window, and apex of the cochlea, which was then bathed in 4% PFA in 0.1 M PB at 4 °C overnight. After decalcification with 0.5 M ethylenediaminetetraacetic acid (EDTA) in PBS [Decalcifying Soln. B (EDTA method); Wako Pure Chemical Industries Ltd., Osaka, Japan] for 7 days at 4 °C with shaking, each cochlea was microdissected into six pieces for whole-mount preparation. Immunostaining was initiated by blocking the tissues for 1 h with 0.1% Triton X-100 in PBS supplemented with 5% goat serum. Fixed and permeabilized pieces were incubated overnight in PBS with a rabbit polyclonal antibody to myosin 7a (1:800; Proteus Biosciences, San Diego, CA, USA) and a mouse monoclonal antibody to CtBP2 (1:400; BD Biosciences, Franklin Lakes, NJ, USA). The samples were washed three times for 20 min each with PBS. Primary antibodies were detected with secondary antibodies conjugated to Alexa 568 for anti-CtBP2 and Alexa 647 for anti-myosin 7a (Invitrogen, Carlsbad, CA, USA). After washing with PBS five times, the specimens were mounted on slides containing an antifade medium (VECTASHIELD; Vector Laboratories, Burlingame, CA, USA) and viewed by confocal fluorescence microscopy (LSM510 Axiovert200M, Carl Zeiss MicroImaging Gmbh, Jena, Germany). The cochlear lengths were measured for each animal, and a cochlear frequency map was computed to precisely localize IHCs from the 10, 12, 16, 20, and 24 kHz regions, *i.e.,* 37.5% (10–12 kHz), 50% (14–16 kHz), 62.5% (18–20 kHz), and 75% (22–24 kHz) distance from apex, respectively. PhotoShop CC (Adobe, San Jose, CA, USA), ImageJ software (NIH, Bethesda, MD, USA), and ImageJ Plugin (http://www.masseyeandear.org/research/otolaryngology/investigators/laboratories/eaton-peabody-laboratories/epl-histology-resources/imagej-plugin-for-cochlear-frequency-mapping-in-whole-mounts) were used to measure the total length of cochlear whole mounts and the length of individual segments. We also referred to the paper relevant to cochlear frequency map^41^.

For frozen cross sections, the rats were perfused transcardially with heparinized PBS followed by 4% PFA in 0.1 M PB at room temperature under general anesthesia. After decapitation, the cochleae were quickly dissected out and bathed in a 4% PFA in 0.1 M PB at 4 °C overnight. Following decalcification with 0.5 M EDTA in PBS for 14 days at 4 °C with shaking, the specimens were cryoprotected in a sucrose gradient (10–30%) and embedded in optimal cutting temperature compound for cryosectioning. Serial frozen sections of 10 μm were cut in the horizontal plane parallel to the cochlear modiolus, stained with hematoxylin and eosin and viewed under a light microscope (BX51, Olympus Corporation, Tokyo, Japan).

### Histopathological analysis

To count the number of OHCs, IHCs, and synaptic ribbons, confocal microscopy was performed at a 37.5% (10–12 kHz), 50% (14–16 kHz), 62.5% (18–20 kHz), and 75% (22–24 kHz) distance from the apex while focusing on the presynaptic ribbons in the basolateral portion of IHCs; an oil-immersion 100× objective and a 0.25-μm z-step were used. For each frequency region, in each cochlea, z-stacks were acquired at three adjacent areas, each containing ~10 IHCs in a row. The number of OHCs and IHCs per 200 μm were counted at each point described above. The densities of OHCs and IHCs per 200 μm were calculated and compared at each site. To further characterize the injury, we labeled afferent synapses using antibodies to CtBP2, a structure associated with synaptic ribbons, which are found on the presynaptic side of the synapse; 95% afferent synapses are associated with IHCs. The numbers of IHC synaptic ribbons (which were identified by punctate labeling) per 200 μm were counted at points 37.5%, 50%, 62.5%, and 75% from the apex as described above. The number of synaptic ribbons per IHCs was calculated and compared at each site. To minimize bias, the counts were performed by three different individuals who were blinded to the experimental groups.

The number of SGCs in the basal, middle, and apical-middle turns of the cochleae was counted. We calculated the average SGC density from the numbers of SGCs obtained from three sections: the section considered to be the center of each frequency site and 20 μm before and after the center section.

### Scanning electron microscopy

For scanning electron microscopy, rats were transcardially perfused under general anesthesia with 0.01 M PB (pH 7.4) containing 8.6% sucrose, followed by fixative consisting of freshly depolymerized 2% paraformaldehyde and 2.5% glutaraldehyde in 0.1 M PB (pH 7.4) containing 5% sucrose. After decapitation, the cochleae were quickly dissected out, and small openings were made at the round window, oval window, and apex of the cochlea, which was bathed in the same fixative at 4 °C overnight. After decalcification with 0.5 M EDTA in PBS (Decalcifying Soln. B (EDTA method); Wako Pure Chemical Industries Ltd.) for 7 days at 4 °C with shaking, each cochlea was microdissected into six pieces for whole-mount preparation. The tissues were then fixed with 1% OsO_4_ at 4 °C for 30 min, dehydrated in ethanol, critical point dried with liquid CO_2_, sputter coated with osmium, and examined with an electron microscope (JSM-6340F, JEOL Ltd., Tokyo, Japan) operated at 5.0 kV.

For a semiquantitative analysis of the stereociliary bundle disruption of OHCs, the ratio of disrupted stereocilia (number of disrupted OHC stereocilia/total number of OHC stereocilia) was calculated from each energy group (n = 3 for each group). The number of stereocilia per 100 μm was counted at the center of the 11-, 16-, and 22-kHz area. “Disrupted” was defined as the outermost row of OHC bundles that were bent at their base toward the lateral side while the other bundle row remained straight. To minimize bias, the counts were performed by three different individuals who were blinded to the experimental groups.

### Statistical Analysis

Statistical analyses were performed using Prism 5 (GraphPad software, Inc., La Jolla, CA, USA). All data values for the ABR thresholds, hair cell counts, and synaptic ribbon counts were compared by two-way ANOVA. Averaged values were compared by one-way ANOVA. P values < 0.05 were considered statistically significant. Error bars represent the standard error (SE) of means.

## Additional Information

**How to cite this article**: Niwa, K. *et al*. Pathophysiology of the inner ear after blast injury caused by laser-induced shock wave. *Sci. Rep.* **6**, 31754; doi: 10.1038/srep31754 (2016).
